# Supplementary material for: Single-Nucleus and Spatial Transcriptomics Revealing Host Response Differences Triggered by Mutated Virus in Severe Dengue
Source: Viruses. 2024 Nov 15;16(11):1779. doi: 10.3390/v16111779 (PMC11599075; doi:10.3390/v16111779)
Supplement: Supplementary file 1 [file viruses-16-01779-s001.zip › viruses-2977713-supplementary.pdf]

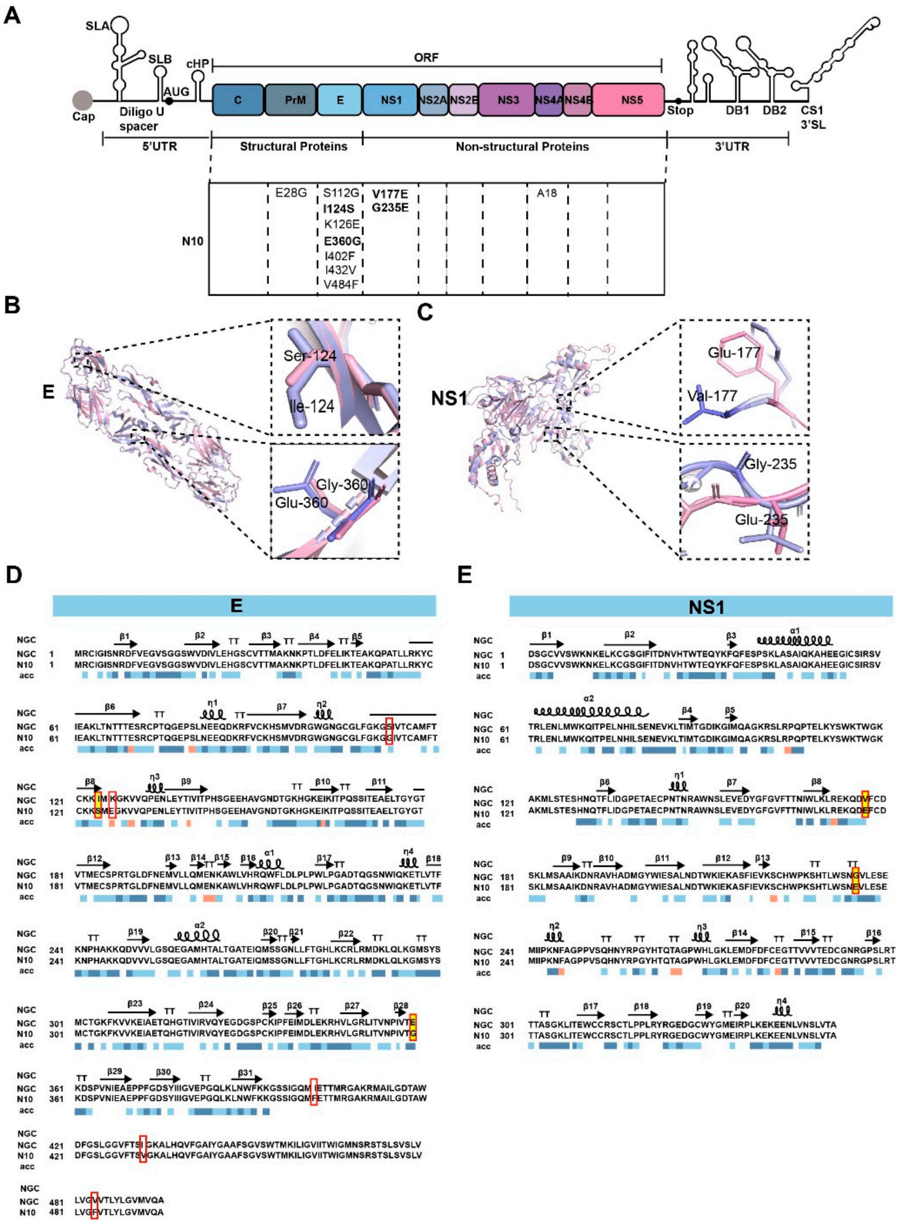


**Supplementary Figure S1.** Cumulative mutations occur in adaptive DENV. (A) The alignment of amino acid sequences between NGC and N10. At the top, a schematic of the DENV genome is shown. (B, C) Tertiary structures of E and NS1 from the NGC and N10 dengue virus strains. (D, E) The secondary structures of the E and NS1 proteins of the NGC and N10 dengue virus strains.

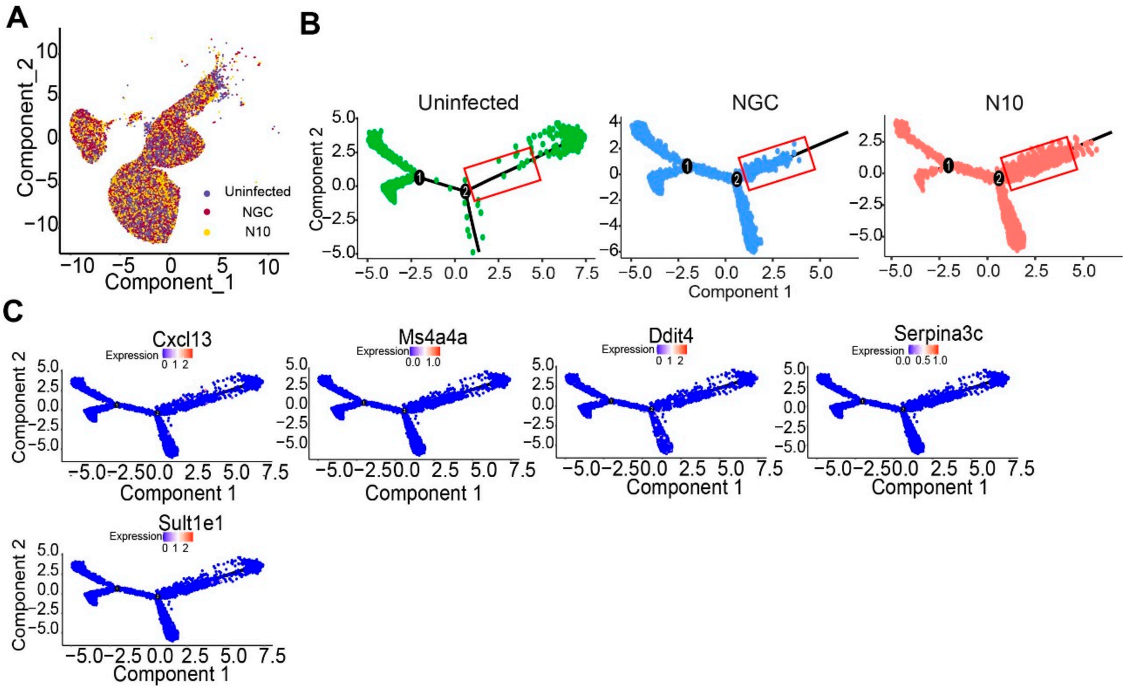


**Supplementary Figure S2.** (A) The UMAP plot of hepatocytes. (B) Pseudo-time analysis of uninfected, NGC and N10 groups. (C) The expression of candidate shared genes of node1 and node2 in the pseudo trajectory.

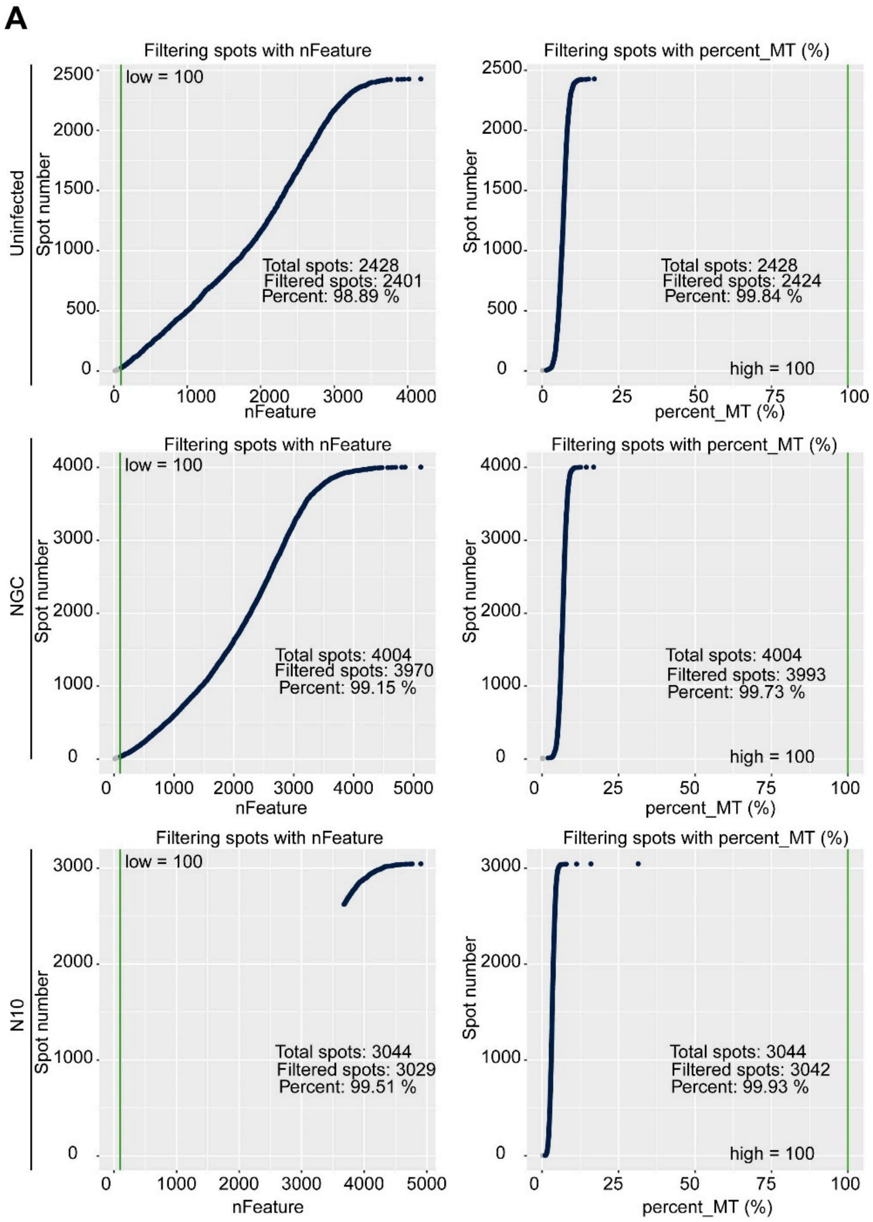


**Supplementary Figure S3. (A).** The number of genes contained in each filtered spots from spatial transcriptome.

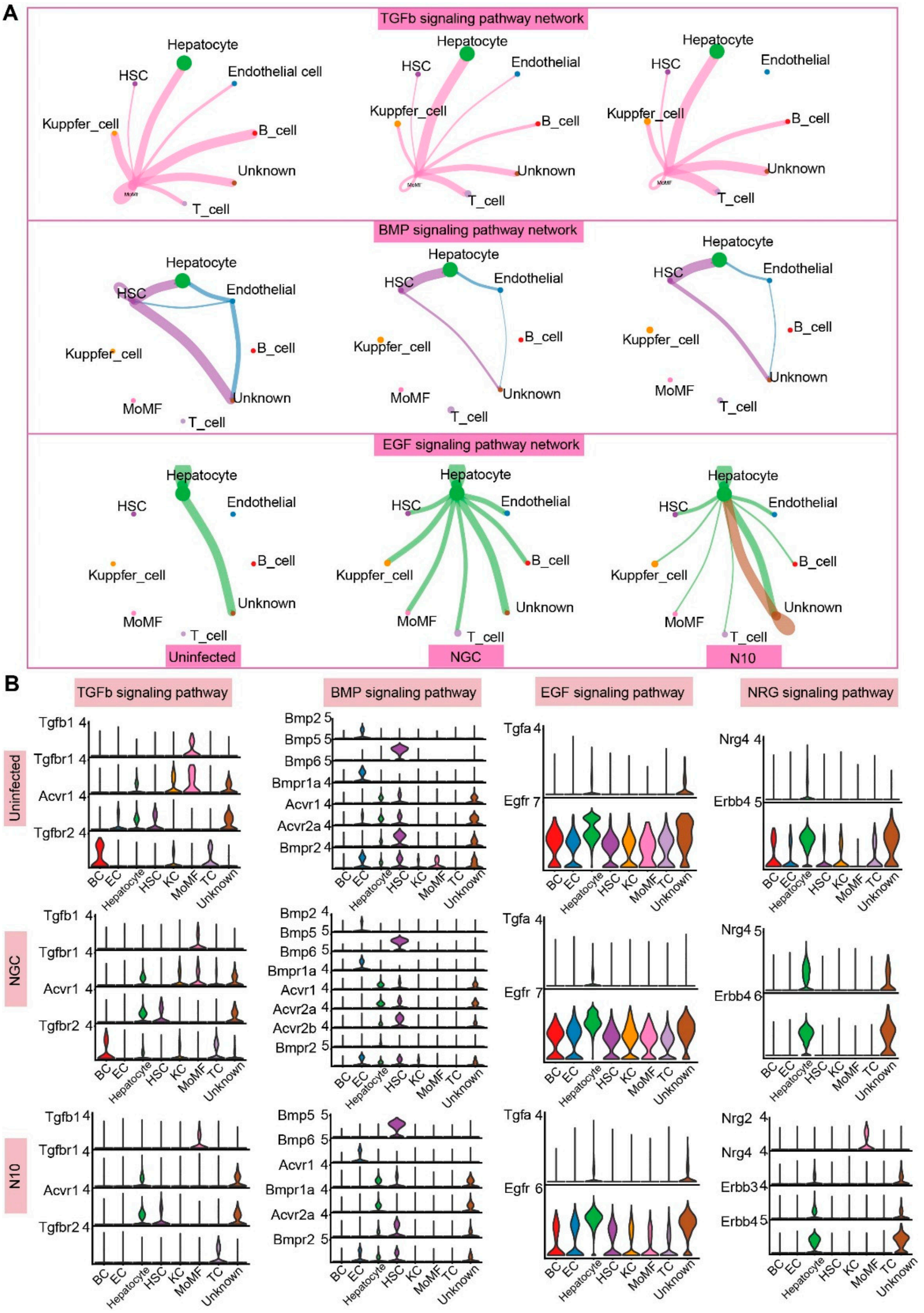


**Supplementary Figure S4.** (A) The TGFb, BMP, EGF signaling pathway network. (B) The gene expression distribution of the TGFb, BMP, EGF and NRG signaling pathway network.

**Supplementary Table S1. Primers for TaqMan qPCR**

| Primers | Upper primer | Lower primer | Probe |
|---|---|---|---|
| DENV2 | GARAGACCAGAGATC CTGCTGTCT | ACCATTCCATTTTCTGGCGTT | FAM-AGCATCATTCCAGGCAC-MGB |

**Supplementary Table S2. Primers for whole genome PCR of N10**

| Primers | Upper primer | Lower primer |
|---|---|---|
| N10 fragment1 | AGTTGTTAGTCTACGTGGACCG | AATGGTCTGATTTCCATCCC |
| N10 fragment2 | CAGACCAGGCTACCATACAC | GTCGCTAAGGTTAAAGCCTC |
| N10 fragment3 | GCCAGGACTCCAAGCAAAAG | CTCAGGGTCAATGCCAGCGCTT |

**Supplementary Table S3. Primers for SYBR qPCR**

| Primers | Upper primer | Lower primer |
|---|---|---|
| Sult1e1 | TGTTGAAATGTTCTTGGCAAGGCC | CATCCTCCTTGCATTTTTCCACATCA |
| Nrg4 | CCTACTATCCCCAGCCCATTCT | TGCCGACAGATTACTTTCGCT |
| Cxcl13 | TGGCTGCCCCAAAACTGA | TGGCACGAGGATTCACACAT |

| | | |
|---|---|---|
| Ddit4 | CTCTTGTCCGCAATCTTCGCT | GGACACCCCATCCAGGTATGA |
| *Mus musculus GAPDH* | ACTCCCACTCTTCCACCTTC | TCTTGCTCAGTGTCCTTGC |
